# Impact of Nutritional and Environmental Factors on Inflammation, Oxidative Stress, and the Microbiome 2019

**DOI:** 10.1155/2019/5716241

**Published:** 2019-07-10

**Authors:** Gang Liu, Yan Huang, Filipa S. Reis, Deguang Song, Hengjia Ni

**Affiliations:** ^1^Hunan Province Key Laboratory of Animal Nutritional Physiology and Metabolic Process, Key Laboratory of Agro-ecological Processes in Subtropical Region, Institute of Subtropical Agriculture, Chinese Academy of Sciences, National Engineering Laboratory for Pollution Control and Waste Utilization in Livestock and Poultry Production, Changsha, Hunan 410125, China; ^2^Department of Animal Science, Division of Agriculture, University of Arkansas, Fayetteville, AR 72701, USA; ^3^Polytechnic Institute of Bragança, Bragança, Portugal; ^4^Yale School of Medicine, New Haven, USA

## 1. Introduction

Metabolically and physiologically, the body is governed by a number of factors: the environment, foodstuffs, the interrelationship and balance of internal microorganisms, and the emotional and physical issues related to stress, inflammation, and illness. Studies show that certain invasive physical and mental issues, possibly caused by surgery, pathogens, or even harmful environmental factors, can lead the body to overgenerate its immune defences, causing inflammation and oxidative stress.

It is known that intestinal microbes react to the immune condition, physiological state, and metabolic activity of their human hosts and that these factors influence health. Extensive but as of yet inconclusive research has identified that probiotics and certain nutrients can assist in the management of oxidative stress, controlling the production of reactive oxygen species (ROS) and reactive nitrogen species (RNS). Some natural compounds and nutraceuticals control the composition of intestinal microbiota, retarding inflammation and allowing the body's natural immune system to control metabolic and physiological processes.

The particular processes that lead to the appearance of disease have yet to be established. The influence of nutrition and the environment, which facilitates the control of inflammation, oxidative stress, and microbiome, requires further study.

This special issue was open to submissions for 9 months from May 2018 to March 2019 and focuses on recent findings on the regulation of inflammation, oxidative stress, and microbiome in diseases and the influence of nutrients, probiotics, and the environment on the development of such diseases.

## 2. Development Mechanisms and Biomarkers of Disease

A. A. Tokmakov et al. focused on the oxidative stress–induced overactivation of* Xenopus* eggs and studied their biochemical hallmarks. Their study showed that time- and dose-dependent overactivation resulted from high levels of hydrogen peroxide–induced oxidative stress. The overactivated eggs were found to have a decreased volume of soluble cytoplasmic protein content, an accumulated volume of lipofuscin, and depletion of intracellular ATP.

There continues to be a major need for tetanus antitoxin (TAT) in developing and underdeveloped countries. It is a comparatively cheap treatment that is easily administered. Nevertheless, there are questions relating to the production of potent TAT using tetanus toxoid (TT) as an immunogen to elicit an immune response. An immunogenicity study was conducted by R. Yu et al. regarding the involvement of the C fragment of tetanus neurotoxin (TeNT-Hc) in the production of TAT. It was found that TeNT-Hc serves as a totally nontoxic recombinant alternative to TT where liver toxicity is not present, with all of the same benefits, as proven in a lengthy safety study. The results showed that TeNT-Hc was suitable for the production of TAT, either separately or in combination with TT.

Y. Wang et al. investigated the necessity for myeloid-derived suppressor cells (MDSCs) and the nuclear transition factor kappa B (NF-KB) for the protective effects of splenectomy, conducted in a mouse model of ConA-induced liver fibrosis. The results showed that whilst the levels of the M2 macrophage inflammatory factors increased after removal of the spleen, the levels of the M1 macrophages and the volume of macrophages/monocytes decreased. Thus, the operation may promote the polarisation of CD11b+Ly6Chigh MDSCs and limit the level of NF-KB p65-p50 heterodimers, which retard the development of liver fibrosis.

Serum and urinary Cr and Fe levels were investigated by Q. Zhou et al., who studied patients in northeast China with impaired fasting glucose (IFG) and impaired glucose tolerance (IGT) and those with type 1 (T1D) and type 2 (T2D) diabetes. They found that serum creatine (serum cr) level decreased in patients with diabetic retinopathy (DR), nephropathy (DN), and peripheral neuropathy (DPN). The highest level of urinary cr was in T1D samples and was considerably higher than those in T2D groups, with or without complications. The urinary Fe level in T1D increased (P < 0.05). Clearly, a positive link existed between serum Cr and serum Fe in T2D patients, indicating that further studies should establish the possible significance of Fe and Cr in diabetes.

Central nervous system cells are protected by leukemia inhibitory factor (LIF) and leukemia inhibitory factor receptor (Lifr), particularly neurons and myelin-sheath oligodendrocytes, in a state of oxygen-glucose deprivation (OGD). L. Huo et al. conducted a study to establish the significance of Lifr in OGD and the mechanism by which it affects hypoxic-ischemic astrocytes. The aim was to derive more information on the neuroprotective role of Lifr. Their findings established the direction and clinical necessity to determine the suitability of Lifr as a repair treatment for neurological and stroke patients.

It has been made increasingly apparent that osteoarthritis (OA) is caused by the degeneration of chondrocytes. Z. Duan et al. showed that parathyroid hormone-related proteins (PTHrP) are targeted by specific microRNAs (miRNAs), which may also control the proliferation and terminal differentiation of chondrocytes. The study further showed that miR-15a-5p was downregulated in OA chondrocytes and that PTHrP was upregulated therein. From these findings, a negative link was established between miR-15a-5p and PTHrP. The reduction of miR-15a-5p encouraged the growth of chondrocytes and restricted calcium build-up, whereas PTHrP was neutralised by the overexpression of miR-15a-5p. These results further clarify the reasons for OA development and signify that miR-15a-5p may be applicable as a biomarker for OA.

V. Kovac et al. conducted a study that examined patients who were undergoing the first 7 days of an orthodontic treatment program with fixed appliances, evaluating various chosen systematic oxidative stress levels. It was found that the treatment may initially lead to the development of systematic oxidative stress, but the effect did not persist. Specifically, increases in ROS and ROS/AD levels were observed only 24 hours after the commencement of treatment. The levels stabilised within 7 days after archwire insertion because of an adaptive endogenous antioxidative response. During subsequent orthodontic treatments, such as archwire reactivation, there was a likelihood that a change of ROS and ROS/AD levels would occur.

Management of oxidative stress in plants has been the subject of several recent studies reviewed by X. Xie et al. This review observed that plants are subject to a number of environmental issues and pressures during their growth, relating to extreme temperature fluctuations, metal toxicity, salinity, and drought. In addition, UV-B radiation, pathogen infection, and pesticides pose challenges to their survival. Plants are able to ameliorate these threats by adapting their molecular, biological, and physiological makeup, in particular via their antioxidant systems. Genetically modified plants, by overexpressing their functional genes, show a marked ability to overcome oxidative stress. This leads to the recommendation that transgenic plants should be cultivated to generate multiple effective genes to combat possibly harmful environments.

A study was performed by F. He et al. to analyse the ligand-independent receptor tyrosine (RTK) cellular signalling pathway and assess its reaction with copper ions (II) (F. He et al.). Copper (II), in the absence of corresponding epidermal growth factor (EGF) and hepatocyte growth factor (HGF) ligands, was used to activate the respective receptor signalling. Two RTK-mediated downstream signal transducers were initiated by copper (II) ions. Proliferation and cellular migration were increased significantly by the use of copper (II). This signifies that cancer treatment may require the growth factors in tumour microenvironments and copper (II) to be points of focus.

## 3. Regulation of Nutritional Factors on Diseases

### 3.1. Botanical Extracts and Natural Compounds

Despite increased research on plant extracts and natural compounds, the impact of these materials on regulating physiological and metabolic development has not been fully analysed and requires further investigation.

Research by Q.-X. Ren et al. focused on the effects of arsenic methylation metabolism and efflux in human hepatocytes (L-02) by proanthocyanidins (PC). Their findings that PC can influence arsenic methylation metabolism and efflux in L-02 cells may account for the increased regulation of glutathione peroxidase (GSH), multidrug resistance protein 1 (MRP1), and arsenite methyltransferase (AS3MT) levels by PC.

The role of chlorogenic acid (CGA), a phenolic secondary metabolite in many fruits and vegetables, was evaluated by S. Peng et al. and assessed for its role in the differentiation and lipolysis of mouse 3T3-L1 preadipocytes. As a peroxisome proliferator-activated receptor-gamma (PPAR*y*2) agonist, CGA can stimulate 3T3-L1 preadipocyte differentiation. It was found that rosiglitazone (RG) performed unlike CGA in relation to fat metabolism in the process of adipocyte differentiation. The RG-treated group contained a markedly higher level of triacylglyceride (TAG) than that observed in the CGA-treated group, possibly because of decreased lipid accumulation before preadipocyte differentiation, due to lipolysis rather than lipid synthesis not being operative. The results show CGA to be a different PPAR*y*2 agonist to RG.

Z. Hong et al. investigated the effects of two dietary additions in an experiment to evaluate the protective effects of certain substances on colitis in mice induced by dextran sodium sulfate (DSS). One preparation was based on an onion preparation fortified only with quercetin aglycone (Q). The second onion preparation contained quercetin aglycone and monoglycosides (Q+MQ). Signs of colitis emerged in mice after 7 days of treatment. The symptoms were colonic inflammation and growth reduction. The results showed that both diets reduced the effects of the DSS-induced colitis, even when administered at a low dosage. A range of fruits and vegetables contain quercetin, and these results indicate that a quercetin-supplemented diet (Q or Q+MQ) is a possible ancillary treatment for inflammatory bowel disorder (IBD).

The* Moringa oleifera* tree, one species of the sole-genus plant family* Moringaceae*, grows in several tropical and subtropical regions. M. Mansour et al. examined the reputed anticancer and antimicrobial attributes of the leaf extract of both* Moringa peregrina* and* Moringa oleifera* grown in Egypt. The serial leaf extract of both varieties displayed antimicrobial characteristics against Gram-positive and Gram-negative bacteria and fungi. The extracts also showed cytotoxic effects against HepG2 and MCF-7 cell lines while displaying low toxicity to normal melanocyte cell lines. Cell cycle arrest and apoptosis of HepG2 cells for anticancer activity analysis were conducted effectively with the diethyl ether and ethyl acetate methods. Analysis via gas chromatography-mass spectrometry analysis showed the leaf extracts to be rich in thymol, retinol and ascorbic, palmitic, linoleic and myristic acids, thereby accounting for the displayed activity.

IBD is exacerbated by dysbiosis and oxidative stress in the gut. The current research on the interaction between gut microbiota and resveratrol, combined with new evidence for the treatment of IBD and oxidative stress, was reviewed by Y. Hu et al. Intestinal health, the cellular redox condition, and the inflammatory response in the host organism are all regulated by gut microbiota. The generation of short-chain fatty acids (SCFAs) and proinflammatory cytokines, linked to enteric bacteria, is able to modulate the proinflammatory NF-kB signalling pathway. Resveratrol and its metabolites are able to reduce increased levels of ROS, activate Nrf2 signalling, and ease oxidative stress by inhibiting inflammatory disorders via changes in the gut microbiota. They act to protect epithelial barrier functions and suppress intestinal inflammation and the activation of NF-*κ*B. Resveratrol is thus a new and effective means to treat chronic inflammatory illnesses.

The antioxidant effects of* Dittrichia viscosa*, with its potential for healing wounds, have been authenticated by W. Rhimi et al. Testing involved processing an ethanolic extract of the leaves with other components to form a mass fraction of 2.5% and 5% extracts in beeswax and sesame oil-based ointments. Analysis of the caffeoylquinic acid content (CQC), the phenol content (TPC), and antioxidant activity in the extract was then performed, and the researchers then conducted a wound-healing test using the composite ointments. It was shown that* D. viscosa* was a source of antioxidant compounds, especially caffeoylquinic acid, and was able to prevent oxidative damage and scavenge free radicals. Treatment of wounded animals using these two ointments resulted in complete repair, with good skin regeneration and re-epithelialization in comparison to other control groups. The study identifies the suitability of* D. viscosa* as an additive to pharmaceutical products to heal wounds and in the treatment of oxidative stress.

The immunity effects of* Orostachys japonicus *(OJ) A. Berger were evaluated by H. Y. Lee et al. in a controlled environment. Mouse splenocytes were treated with either extract of OJ or water, both with and without cyclophosphamide (CY). In the presence of OJ extract, an increase was seen in the propagation of splenocytes, and a reduction was seen in CY-induced cytotoxicity. Immunosuppressed rats displayed improved stamina after treatment with OJ extracts when assessed with an obligatory swim test. Treatment with the extract increased the number of immunity-related cells and the levels of plasma cytokines. Thus there is a clear indication that OJ treatment increases immune cell propagation and specific plasma cytokine levels, thereby improving immunity.

An innovative cross-linked omics study was conducted by T. Li et al. The aim was to evaluate the effect of myricetin, a common flavonoid found in many edible plants. They evaluated its anti-inflammatory properties by inspecting the molecular evidence and performing a proteomics assay of gene expressions in a genome-wide analysis, utilising microarrays and specialist analysis tools. The results showed that myricetin exerted a significant anti-inflammatory effect on metabolic disease and lipid metabolism in HepG2 cells and on cardiovascular disease. The indications are that myricetin could have future pharmaceutical health applications. Well-structured research, combining anti-inflammatory proteomics analysis and gene chip data, has led to improved considerations for its multiomic use.

### 3.2. Probiotics

Probiotics have wide-ranging applications in medical science. Alone or in combination with prebiotics, they are instrumental in controlling gut microbiota, thereby displaying anticancer, angiogenic activities, antipathogenic, antiobesity, diabetic and antidiabetic, anti-inflammatory, and angiogenic activities, in addition to their positive results on the central nervous system and the brain.

M. A. K. Azad et al. reviewed recent research on the effects of probiotic immunomodulators and found that their antiviral properties influenced innate immunity. By stimulating B cells, they improved gut barrier functions and influenced cytokine production, thereby generating host body adaptive responses. More research is needed to establish how probiotics create immunomodulatory effects to combat inflammation.

F. Zhang et al. conducted an experiment using* Lactobacillus plantarum* to investigate its effect on gut inflammation and its interaction with gut microbiota and the immune response. Mice that had been treated with DSS to initiate colitis were fed* Lactobacillus plantarum*. This markedly lowered the production of proinflammatory cytokines during the colitis development phase, and the results indicated that the treatment could improve the pathophysiology of DSS generated colitis. The impact of intestinal microorganisms was lower in the* L. plantarum* group than in the DSS group, showing improved tract stability. The indications are that* L. plantarum* could be used as an IBD treatment because it can manage the symptoms of colitis.

### 3.3. Other Nutrients

Anti-inflammatory effects are apparent in IRW (Ile-Arg-Trp), which is a bioactive peptide isolated from egg ovotransferrin. An investigation of IRW for use in the treatment of inflammatory cytokines and microbiota was conducted by H. Jiao et al. IRW lowered the serum levels of tumour necrosis factor- (TNF-) a, interleukin- (IL-) 6, and myeloperoxidase (MPO) activity in a lipopolysaccharide- (LPS-) induced rat model. There was an increase in the Shannon and a decrease in the Simpson indices of faecal microbiota. IRW treatment noticeably limited the LPS-enhancement of TNF-a, IL-8, vascular cell adhesion molecule-1 (VCAM-1), and intercellular cell adhesion molecule-1 (ICAM-1) in human umbilical vein endothelial cells (HUVECs). These results indicate that bioactive peptides can be used in anti-inflammatory treatments.

X. Mao et al. investigated the effects of benzoic acid on gut functions. The acid is used as an organic acidifier and is found in a variety of foodstuffs, where it acts as an antifungal and antibacterial preservative. By regulating redox status, immunity, enzyme activity, and microbiota, the acid can aid digestion, barrier, and gut absorption functions. Used as a supplement in foodstuffs, it is particularly effective in improving convalescent health. A benzoic acid dosage of 0.2 to 0.5% is recommended in foodstuffs.

A study was conducted on nutritional cytokine leptin produced by cancer-associated fibroblasts (CAFs) with the aim to study their paracrine effects and mechanisms. Because leptin is the main component of the tumour microenvironment in nonsmall cell lung cancer (NSCLC), a novel* in vitro* cell coculture system was established. F. Li et al. discovered that leptin produced by CAFs can induce the proliferation and migration of NSCLC cells. This probably occurs via the P13K/AKT and MAPK/ERK1/2 paracrine intracellular-signalling pathways. This study indicates that nutritional factors are significant in tumour–tumour microenvironment interactions and suggests there may be a path towards the treatment of NSCLC.

S. Huang et al. investigated the benefits of milk fat globule membrane (MFGM), a protein-lipid complex that surrounds the fat globules in milk. Beneficial for the health of animals, it was seen to enhance the growth rate of low birth weight neonatal (LBW) mice, particularly during their early life. In addition, it relieved intestinal damage incurred from LPS challenge in LBW mice by increasing the messenger RNA (mRNA) levels of tight junction proteins, antioxidant enzymes, and intestinal mucosal barrier proteins. Inhibition of TLR2 and TLR4 signaling reduced the expression of proinflammatory cytokines. These indications showed that MFGM is a beneficial nutrient for the enhancement of growth performance, creating a novel means for the prevention and treatment during the early stages of intestinal inflammation in LBW neonates.

M. Wiese et al. performed an interesting clinical study on moderately obese persons, comprising 15 men and 15 women within an age bracket of 55 ± 5.7 years, to establish the prebiotic effect of lycopene and dark chocolate. These moderately obese subjects (30<BMI < 35 kg/m^2^) undertook a 1-month trial, and the systematic effects of lycopene or dark chocolate intake were recorded at the end of the trial period. The results were improved blood, gut, and liver lipid metabolism, plus improvements in skeletal muscle and skin conditions. These were deduced to have not been solely a result of the properties of the carotenoid and chocolate, but likely also due to modulation of the gut microbiome, which increased the presence of* Lactobacilli* and* Bifidobacteria* and thus their considerable beneficial effects.

Q. Jiang et al. summarised recent studies of antiquorum sensing (QS) agents, together with their signals in response to pathogens, highlighting the possibility of QS therapy in the treatment of bacterial diseases. Studies have identified many anti-QS agents that can control the pathogenic phenotypes of many types of bacteria. These agents have been shown to limit the pathological damage in infected animal models. They lack the stability of antibiotics and possibly have toxicity, which limits their widespread usage, but when combined with conventional antibiotics they are shown to enhance their effectiveness at a reduced cost, indicating their potential for future bacterial disease treatment.

This issue aims to focus readers' attention on the latest scientific work that is being conducted in relation to nutrients, inflammation, the environment, stress, and microorganisms, highlighting some of the innovative research methods used.

